# Locoregional Treatment of Bone Metastases in a Lung Cancer Patient: A Case Report Using Multiple Techniques: Electrochemotherapy, Cryoablation, and Cementoplasty

**DOI:** 10.3390/reports9020172

**Published:** 2026-06-01

**Authors:** Francesco Fiore, Salvatore Stilo, Luca Tarotto, Emanuela Federico, Noemi Brignola, Gaetano Sicuranza, Roberto D’Angelo

**Affiliations:** 1 Division of Interventional Radiology, Istituto Nazionale per lo Studio e la Cura dei Tumori “Fondazione G. Pascale”, IRCCS of Naples, 80131 Naples, Italy; salvatore.stilo@istitutotumori.na.it (S.S.); luca.tarotto@istitutotumori.na.it (L.T.);; 2 Division of Radiology, Università Degli Studi di Napoli Federico II, 80131 Naples, Italy; emanuela.federico@studenti.unina.it (E.F.); noemi.brignola@studenti.unina.it (N.B.); 3 Division of Radiology, AOU Città Della Salute Torino, 10126 Torino, Italy

**Keywords:** electrochemotherapy, bone metastases, pain, quality of life

## Abstract

**Background and Clinical Significance**: Bone involvement is a common and debilitating manifestation of advanced malignancies, with a substantial negative impact on patients’ functional status, quality of life, and overall prognosis. Management is primarily palliative and may include several locoregional approaches such as radiotherapy, surgical stabilization, cementoplasty, thermal or cryoablation, and high-intensity focused ultrasound. Electrochemotherapy (ECT) is an emerging non-thermal ablative technique that combines limited invasiveness with short procedural times and a favorable safety profile. **Case Presentation**: We report the case of a patient with oligometastatic lung cancer presenting with a painful rib metastasis refractory to radiotherapy. The patient had previously undergone radiotherapy to the right femoral head and the eighth rib, followed by cryoablation combined with cementoplasty for the femoral lesion and cryoablation of the rib. At one-year follow-up after cryoablation combined with bone cementoplasty, computed tomography demonstrated progression with the appearance of a new symptomatic rib lesion unresponsive to further radiotherapy. Percutaneous ECT was therefore performed under general anesthesia, supplemented with an erector spinae plane block. A total of twelve 18-gauge needle electrodes were accurately positioned under fluoroscopic guidance. Follow-up imaging at three months showed complete local tumor resolution, accompanied by marked and sustained pain relief. **Conclusions**: This experience supports the role of ECT as an effective salvage locoregional treatment option in selected patients with bone metastases resistant to conventional therapies.

## 1. Introduction and Clinical Significance

Electrochemotherapy (ECT) is a therapeutic approach that integrates the administration of a cytotoxic agent with electroporation (EP). This technique involves the delivery of short, high-voltage electric pulses to the cell membrane, inducing a transient increase in membrane permeability. As a result, molecules that are typically non-permeant or only minimally permeant can enter the cytoplasm through diffusion or active transport mechanisms. Following the initial clinical trials of electrochemotherapy [1,2,3,4] and the subsequent establishment of standardized operating procedures [4,5,6,7], a growing body of evidence has consistently demonstrated its clinical efficacy along with a favorable safety profile.

The effectiveness of both ECT and irreversible electroporation (IRE) in the management of bone metastases has been well documented [8,9,10,11,12,13,14,15]. In particular, ECT has been shown to achieve significant pain reduction and to enhance patients’ quality of life, without causing major complications [16,17,18,19]. Moreover, ECT has proven to be a safe and feasible treatment option for painful bone metastases, including cases in which prior therapeutic strategies have failed [20]. Early experiences with ECT in the treatment of spinal metastases yielded promising outcomes [11]. These findings were later supported by studies demonstrating the feasibility and efficacy of image-guided percutaneous ECT in spinal lesions [13]. Additionally, the safety and therapeutic effectiveness of percutaneous ECT have been confirmed in patients with radiotherapy-resistant metastatic epidural spinal cord compression (MESCC) [15].

In the present report, we describe the three-month clinical outcome following ECT treatment in a patient for whom no further therapeutic options were available.

## 2. Case Presentation

**Figure 1:**
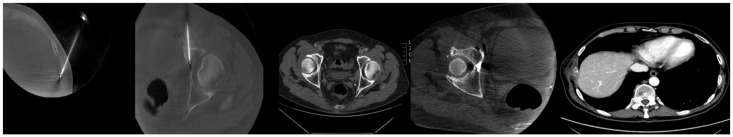
Cryotherapy and cementoplasty of the hip and cryoablation of the eighth rib.

A 61-year-old male patient, previously treated with targeted therapy for a primary left lung adenocarcinoma developed skeletal disease progression two years after the initial diagnosis. The patient subsequently underwent radiotherapy (RT) to the right femoral head and the eighth rib. Due to persistent right hip pain associated with significant functional impairment, the case was discussed by a multidisciplinary oncologic team, which recommended locoregional treatment. Systemic assessment was performed by both contrast-enhanced CT and total-body PET-CT. PET-CT. The patient therefore underwent cryoablation combined with cementoplasty of the right hip lesion and cryoablation of the eighth rib metastasis (Figure 1). At presentation, the patient’s Eastern Cooperative Oncology Group (ECOG) performance status was 1, with oligometastatic skeletal disease involving the right femoral head and the right eighth rib.

**Figure 2:**
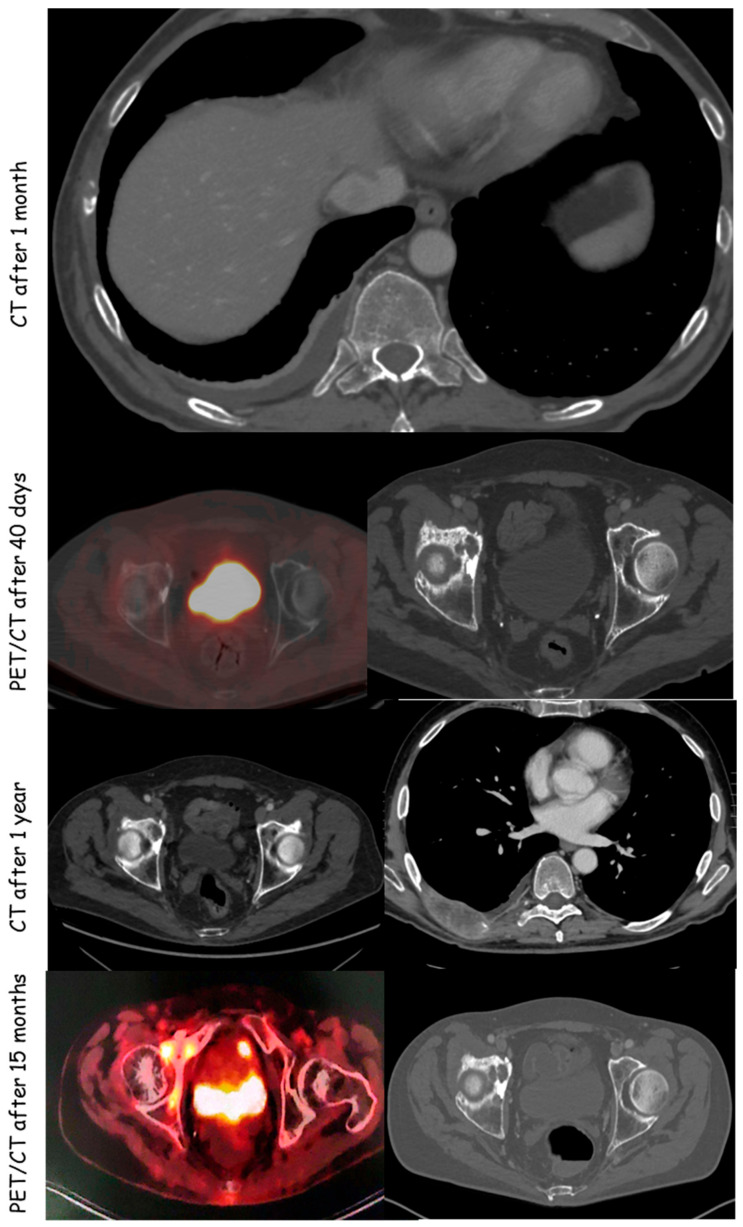
CT at 1 month and at 1 year from the treatment of cryotherapy with cementoplasty. PET/CT at 40 days and 15 months from above treatment.

CT scan at 1 year, revealed a painful rib metastasis resistant to RT (Figure 2).

Electrochemotherapy was selected as the salvage treatment strategy given the failure of prior radiotherapy and the technical limitations precluding further cryoablation at this site, combined with the institutional expertise in ECT for bone metastases. Prior to the procedure, the patient reported a pain score of [8/10] on the Numerical Rating Scale (NRS), with an analgesic regimen consisting of (details of the analgesic regimen at the time of ECT were not available from the medical records).

After providing written informed consent, the patient underwent electrochemotherapy (ECT). The procedure was carried out under general anesthesia combined with an erector spinae plane (ESP) block. The patient was positioned prone, and cone-beam computed tomography (CBCT) was performed to accurately localize the known metastatic lesion involving the right eighth rib. CBCT was performed using standard acquisition parameters (120 kV, 100 mAs, 25 cm field of view, 1 mm slice thickness reconstruction). Post-procedural imaging response was assessed on contrast-enhanced CT, defining complete tumor necrosis as the complete replacement of the pre-treatment enhancing lesion by non-enhancing hypodense tissue, consistent with devascularisation, in accordance with the criteria adopted in previously published ECT bone metastasis studies [20,21,22,23,24,25,26,27,28,29,30].

Following sterile preparation and local anesthesia, a total of twelve 18-gauge needle electrodes were inserted under fluoroscopic guidance. The electrodes were placed in a parallel configuration, targeting the deepest portion of the lesion.

Bleomycin was administered intravenously at a dose of 15,000 IU/m^2^ prior to pulse delivery. Electric pulses were subsequently applied using multiple single-needle electrodes arranged in a variable geometry configuration (VGD applied part; IGEA Ltd., Modena, Italy). Pulse delivery was performed using the Cliniporator™ system (IGEA Ltd., Modena, Italy), with parameters set according to the ESOPE (European Standard Operating Procedures of Electrochemotherapy) guidelines [9,16]: eight pulses per electrode pair, an electric field intensity of 1000 V/cm, and a pulse duration of 100 μs.

To ensure procedural safety, pulse delivery was synchronized with the patient’s electrocardiogram. ECG synchronization was achieved using the Norav 1220T system (medical device provided by IGEA S.p.A., Carpi, Italy). The treatment was completed within the recommended therapeutic window, between 8 and 40 min following the completion of the bleomycin bolus administration [17].

**Figure 3:**
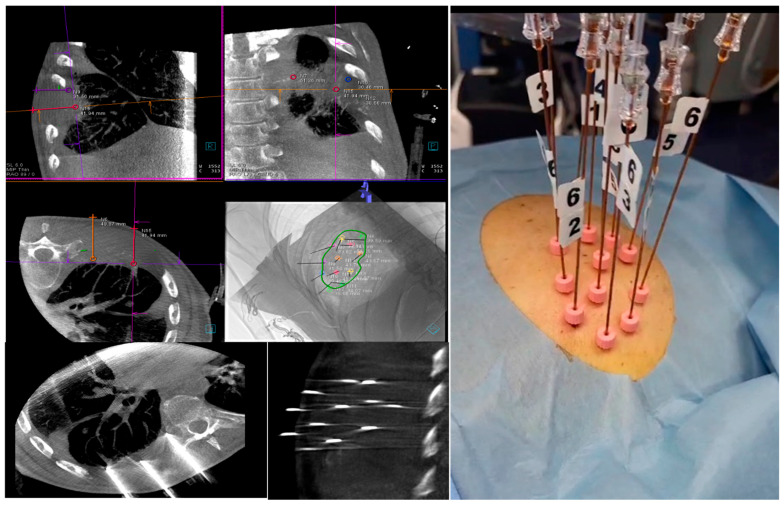
ECT planning and needle positioning under CBCT guidance.

Electrode placement and treatment geometry were defined using a dedicated preoperative planning software. Each single needle electrode featured an active length of 30 mm and a total length of 20 cm (Figure 3).

CBCT showed no peri- and post-procedural complications. The needles were removed and the patient was subjected to medication. Supine decubitus for at least 5 h and resting for 24 h was required.

CT was performed one day and three months after the ECT treatment.

## 3. Results

**Figure 4:**
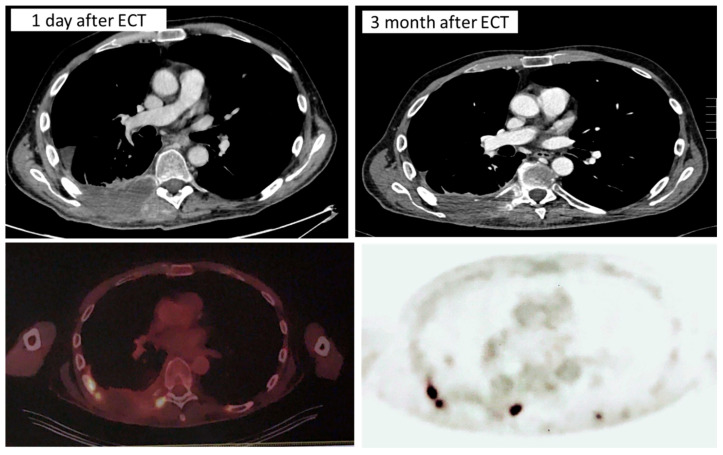
ECT evaluation by imaging.

Post-procedural CT performed one day after ECT (Figure 4), acquired with intravenous contrast medium, demonstrated right-sided hydropneumothorax with associated compressive atelectasis, as a post-procedural finding likely attributable to the intercostal ECT approach, targeting tissue between the eighth and ninth ribs, with involvement of the right paravertebral musculature and extension to the pleural margin. No intracranial lesions or laterocervical lymphadenopathy were identified. The hydropneumothorax was managed conservatively; specific details of the management protocol and clinical outcome were not available from the medical records.

At the three-month follow-up CT and PET-CT scan, the posterior arch of the right eighth rib appeared completely replaced by hypodense tissue over approximately 8 cm, showing no vascularization, consistent with complete tumor necrosis replacing the pre-ECT solid mass (Figure 4). PET-CT confirmed the markedly reduced metabolic activity at the treated site, corroborating the local imaging response. These imaging findings corresponded to significant clinical improvement, with a pain score reduction from 8/10 to 2/10 and a reduction in analgesic consumption. The patient’s ECOG performance status remained stable at 1 at the three-month follow-up. Specific changes in the analgesic regimen were not documented in the available medical records.

However, PET-CT demonstrated systemic disease progression, with an increase in the hepatic lesion and the appearance of new osteosclerotic lesions at the D10 vertebral body and multiple sclerotic lesions involving the pelvis and sacrum bilaterally, the largest measuring 22 mm at the right sacroiliac level. Figure 4 CT and PET-CT scan at one day (left panels) and three months (right panels) after the ECT treatment.

## 4. Discussion

Bone metastases constitute a frequent and severely debilitating complication in patients with cancer, with a substantial detrimental impact on overall health status, quality of life, and survival. Improvements in systemic oncologic treatments have extended patient life expectancy, which has been accompanied by an increased incidence of metastatic dissemination [18,19]. The skeleton represents the third most common site of metastasis and may be involved early in the disease course, particularly in breast, lung, and prostate malignancies.

Pain associated with bone metastases arises from multiple mechanisms, including cytokine-mediated nerve activation, periosteal stretching, compression of adjacent soft tissues and neural structures, and, in some cases, pathological fractures [30,31,32]. Clinically relevant pain is reported in approximately 70% of patients with skeletal metastases [20], making pain control a primary therapeutic goal. In addition, bone metastatic disease is frequently complicated by fractures, spinal cord compression, hypercalcemia, and impaired mobility, all of which significantly increase patient morbidity.

Several palliative treatment options are currently available for metastatic bone disease, including radiotherapy, thermal ablation techniques such as radiofrequency ablation and cryoablation, high-intensity focused ultrasound, surgical resection, and cement augmentation of osteolytic lesions. When surgical or ablative approaches are contraindicated, pain management may rely on systemic analgesics, including opioids, as well as minimally invasive techniques such as radiofrequency ablation or selective embolization [9]. Treatment selection is guided by tumor histology and disease stage, with the principal objectives of pain relief, fracture prevention or management, and improvement of quality of life.

Over the last decade, the evolution of interventional radiology has profoundly influenced the local management of bone metastases, representing one of the most relevant advancements in this field [33]. Interventional radiologic approaches aim to achieve three main goals: local tumor control, analgesia, and reduction in fracture risk. Each technique is associated with specific indications in both oncologic and palliative settings [34]. Prior to any image-guided intervention on skeletal lesions, assessment of fracture risk is mandatory, and prophylactic stabilization should be considered in cases of high risk, even in asymptomatic patients [35].

Cementoplasty may be performed as a standalone procedure or in combination with other locoregional therapies [36,37]. Its purpose is the percutaneous injection of bone cement into osteolytic cavities under imaging guidance, with the aim of restoring structural stability and alleviating pain [38,39]. Pain relief following cementoplasty is often durable and is attributed to both mechanical reinforcement and local tumor necrosis induced by the cement [34]. Experimental and clinical studies suggest that cement-related necrosis is largely limited to the cemented area and does not extend beyond approximately 5 mm, likely due to the high temperatures generated during cement polymerization (70–80 °C) [38].

The technique consists of injecting polymethylmethacrylate (PMMA) into fractured or structurally weakened bone segments infiltrated by tumor tissue, thereby reinforcing the bone and reducing mechanically induced pain. PMMA delivery is performed under CT or fluoroscopic guidance directly into the lesion [40,41]. Cementoplasty is particularly indicated in load-bearing anatomical regions such as vertebral bodies, the acetabulum, and the proximal or distal epiphyses of long bones [42]. As cementoplasty primarily provides mechanical stabilization and analgesia rather than direct tumor eradication, it is frequently combined with additional tumor-targeted therapies [43].

Cryoablation is a minimally invasive thermal ablation technique that induces tumor destruction through exposure to extremely low temperatures. One or more probes delivering liquid nitrogen or compressed gas are inserted into the lesion under imaging guidance [44]. Repeated freeze–thaw cycles result in tumor cell death through intracellular ice crystal formation, vascular injury, ischemia, and reduced perfusion [45]. Temperatures below −20 °C produce complete cellular necrosis, whereas exposure to temperatures between 0 °C and −20 °C leads to destruction of approximately 80% of tumor cells [46].

Electrochemotherapy (ECT) is a non-thermal, minimally invasive local treatment characterized by short procedural times and a low rate of severe adverse events. Preclinical studies have demonstrated that ECT preserves bone mineral structure while showing promising therapeutic potential in skeletal lesions [8,11,26]. Clinically, ECT has been shown to be safe and effective for the treatment of bone metastases and may be used either alone or in combination with radiotherapy [27,28,29,30,31,32,33,34,35,36,37,38,39]. Several reports have described its application in vertebral metastatic disease [11,13].

A clinical trial conducted at the Istituto Ortopedico Rizzoli (Bologna, Italy) evaluated the feasibility and safety of ECT in patients with bone metastases, confirming a favorable safety profile and rapid postoperative recovery [9,12]. Campana et al. further demonstrated that ECT represents a safe and feasible option for the treatment of painful bone metastases, including cases refractory to previous therapies [25].

ECT has also been successfully applied in spinal metastases involving the posterior wall of lumbar vertebral bodies, with cone-beam computed tomography guidance used to enhance procedural safety. In these scenarios, ECT proved to be a valuable alternative when other treatment modalities were unsuitable, resulting in significant pain reduction, improvement in quality of life, and favorable local tumor control [27].

Cindric et al. described a transpedicular approach combining ECT with established spinal fixation techniques for the treatment of vertebral metastases [30]. In this method, needle electrodes were advanced through the pedicles and positioned circumferentially around the tumor within the vertebral body. The technique was found to be safe and feasible when the majority of tumor volume was confined to the vertebral body, and effective treatment was achievable even when tumor margins were in close proximity to the spinal cord or nerve roots.

Deschamps et al. [15] assessed the safety and efficacy of percutaneous ECT in a cohort of 40 patients with radiotherapy-resistant metastatic epidural spinal cord compression (MESCC). Seventeen-gauge needle electrodes were inserted percutaneously via bone access needles in a hybrid operating room equipped with fluoroscopic and CT imaging. At a median follow-up of 5.1 months, pain improvement was reported in 80% of patients at one month and persisted in 67% at three months. Neurological improvement was also observed. Among the 35 patients evaluable at one month, complete response was achieved in 46%, partial response in 31%, and stable disease in 23%, with no cases of disease progression. At three months, complete response persisted in 28.5% of patients, while partial response, stable disease, and progression were observed in 38%, 24%, and 9.5% of cases, respectively. Overall tumor response at one month was significantly higher in lesions involving one or two vertebral levels and in hypervascular tumors compared with hypovascular lesions.

In the present study, persistent skeletal metastases following an initial course of radiotherapy prompted the use of additional locoregional treatments, including cryoablation to induce tumor ischemia and cementoplasty to achieve mechanical stabilization and cement-related tumor necrosis. Despite these interventions, the development of new metastatic lesions on follow-up CT imaging required consideration of an alternative therapeutic strategy due to lesion location and extent. Electrochemotherapy was therefore selected and performed via percutaneous electrode insertion according to a predefined geometry and patient-specific treatment plan, ensuring adequate electroporation of the metastatic tissue. Post-treatment CT imaging performed 24 h after the procedure demonstrated reduced lesion density consistent with early tumor response and no procedure-related complications, as assessed using the Common Terminology Criteria for Adverse Events (CTCAE). At the three-month follow-up, the patient demonstrated a clinically significant pain response, with NRS scores decreasing from 8/10 to 2/10, alongside sustained local tumor control documented on CT and PET-CT imaging, reduced analgesic requirements (specific changes in the analgesic regimen were not documented in the available medical records), and a notable improvement in functional status.

Consistent with published criteria, severe adverse events were defined as those requiring major medical intervention [12].

Several inherent limitations of this report must be acknowledged. First, as a single-case observation, the findings cannot be generalized to a broader patient population, and the conclusions drawn from this experience must be interpreted with caution. Second, the absence of quantitative pre- and post-procedural pain assessments using validated instruments (e.g., Numerical Rating Scale or Visual Analog Scale) limits the objective evaluation of clinical benefit. Third, follow-up duration was limited to three months for local disease control; longer-term data are needed to assess the durability of the locoregional response. Despite these limitations, this case contributes to the growing body of evidence supporting ECT as a viable salvage option for radiotherapy-resistant bone metastases and may inform the design of future prospective studies.

## 5. Conclusions

Locoregional therapies should be systematically integrated into the multidisciplinary management of oncologic patients, including those with oligometastatic disease. These approaches offer high rates of local tumor control, and the selection of the most appropriate technique for each clinical scenario requires the expertise of an experienced interventional radiologist. In patients with metastatic lesions refractory to radiotherapy, locoregional treatments deserve careful consideration to achieve effective pain palliation and sustained local disease control.

Among the available strategies, cryoablation and cementoplasty represent valuable options for the treatment of bone metastases, as they provide mechanical stabilization of osteolytic lesions while promoting local tumor necrosis.

Electrochemotherapy (ECT) has recently emerged as a safe and feasible locoregional treatment for painful skeletal metastases and should be considered a therapeutic alternative even in patients who have failed previous treatment lines.

## Data Availability

The original data presented in this study are available on reasonable request from the corresponding author. The data are not publicly available due to privacy concerns.
